# The Popeye domain containing gene family encoding a family of cAMP-effector proteins with important functions in striated muscle and beyond

**DOI:** 10.1007/s10974-019-09523-z

**Published:** 2019-06-13

**Authors:** Alexander H. Swan, Lena Gruscheski, Lauren A. Boland, Thomas Brand

**Affiliations:** 1 National Heart and Lung Institute, Imperial College London, 4th Floor ICTEM Building, Du Cane Road, London, W12 0NN UK; 2 Institute of Chemical Biology, Imperial College London, London, UK

**Keywords:** Popeye domain containing genes, cAMP, Sinus node disease, Atrioventricular block, Limb-girdle muscular dystrophy, Tumour suppressor, Membrane trafficking

## Abstract

The Popeye domain containing (POPDC) gene family encodes a novel class of membrane-bound cyclic AMP effector proteins. POPDC proteins are abundantly expressed in cardiac and skeletal muscle. Consistent with its predominant expression in striated muscle, *Popdc1* and *Popdc2* null mutants in mouse and zebrafish develop cardiac arrhythmia and muscular dystrophy. Likewise, mutations in POPDC genes in patients have been associated with cardiac arrhythmia and muscular dystrophy phenotypes. A membrane trafficking function has been identified in this context. POPDC proteins have also been linked to tumour formation. Here, POPDC1 plays a role as a tumour suppressor by limiting c-Myc and WNT signalling. Currently, a common functional link between POPDC’s role in striated muscle and as a tumour suppressor is lacking. We also discuss several alternative working models to better understand POPDC protein function.

## Introduction

In 1999 the first member of the Popeye domain containing (POPDC) gene family was cloned in the chicken (Reese et al. 1999), which was shortly followed by the isolation of the human gene (Reese et al. 1999). These authors named this gene blood vessel epicardial substance (*Bves*) based on the observed preferential expression of Bves protein in the developing epicardium and subsequently in the coronary vasculature (Hager et al. 2010). A family of three isoforms, including Bves (which was named Pop1 by these authors) was independently isolated from the chicken, mouse and human heart and named as Popeye (POP) genes based on their preferential expression in striated muscle (skeletal and cardiac muscle) (Andrée et al. 2000). Due to the presence of genes already named Pop, which encode ribonuclease proteins, the family was renamed as the Popeye domain containing genes consisting of *Popdc1 (Bves)*, *Popdc2* and *Popdc3* (Andrée et al. 2002b; Brand 2005). These names will be utilized throughout this article.

The disparate expression pattern described for Popdc1 is probably based on off-target recognition of antigens in the epicardium and vessel wall by some of the antibodies previously employed (Osler et al. 2006; Wada et al. 2001). A hallmark of POPDC genes in vertebrate species is a strong expression in heart and skeletal muscle, which was demonstrated in multiple species using several different approaches i.e. Northern blot, RT-PCR, in situ hybridization, β-galactosidase (LacZ) staining and also by immunohistochemistry using antibodies, which were validated to be non-reactive with null mutant tissue (Brand 2005; Froese et al. 2012; Schindler et al. 2016). Apart from a strong expression in striated muscle, POPDC gene expression is also found in other cell types and organs. For example, POPDC genes are expressed in the smooth muscle tissue of the digestive tract, bladder, uterus and lung, central and peripheral neurons and some epithelial cells (Andrée et al. 2000, 2002a; Froese and Brand 2008).

In all vertebrates, three genes are localised on two different chromosomes. *POPDC1* and *POPDC3* in men are localized in a tandem configuration on chromosome 6q21, while *POPDC2 is* localized to chromosome 3q33.33 (Andrée et al. 2000; Brand 2005). In the human genome and in large mammals, the intergenic region between *POPDC1* and *POPDC3* contains a gene for a long noncoding RNA called *BVES*-*AS1*; at present however, no genetic or functional interaction with either *POPDC1* or *POPDC3* has been described. Interestingly, *POPDC1*, *POPDC3* and *BVES*-*AS1* have been linked to colon adenocarcinoma (Luo et al. 2019; Williams et al. 2011). Moreover, *BVES*-*AS1* is expressed at high levels in heart and skeletal muscle, suggesting co-regulation with *POPDC1* and *POPDC3* (Genevisible 2016). Unfortunately, *BVES*-*AS1* is not present in the murine genome and therefore its role cannot be studied in this species.

## The cAMP signalling pathway

**Fig. 1:**
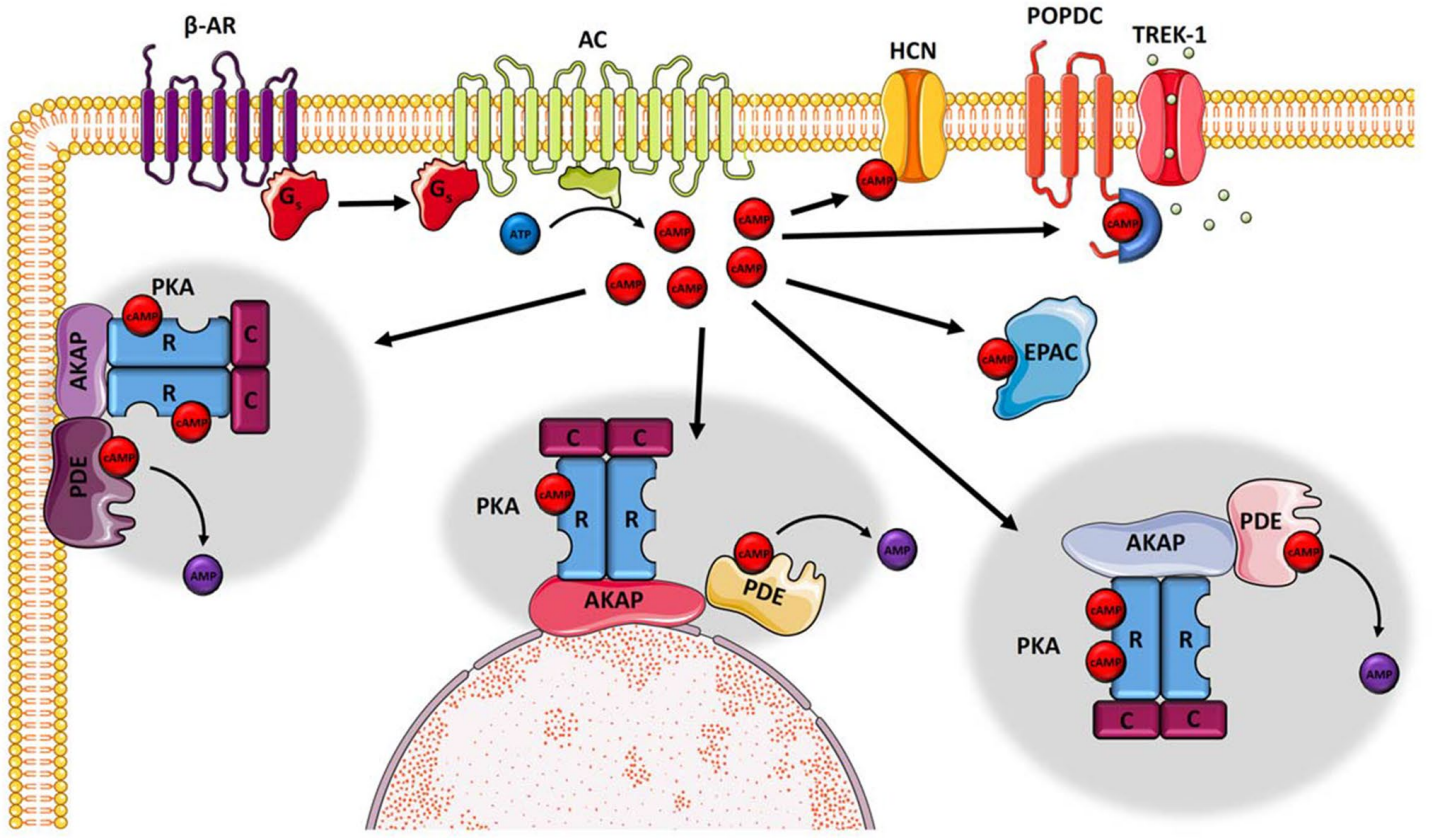
The cAMP signalling pathway. Activation of adenylyl cyclase (AC) by G-coupled receptors (GPCR) via Gs leads to an accumulation of cAMP, which is bound by a number of different cAMP effector proteins, namely HCN channels and POPDC proteins, which are both membrane-bound, while EPAC and PKA are cytoplasmic proteins. PKA is bound by a diverse group of anchor proteins (AKAP), which also recruit PDE isoforms forming a nanodomain. PKA activation and substrate phosphorylation is under tight spatiotemporal control. There are a large number of AKAP proteins in cells creating many different cAMP nanodomains in different subcellular compartments (grey halo). AKAP proteins also bind other signalling molecules forming a platform also allowing cross-talk between signalling pathways

3′-5′-cyclic adenosine monophosphate (cAMP) is a universal second messenger that is important in mediating many cellular responses. It has been shown to accumulate in cardiac myocytes upon β-adrenergic stimulation and is therefore an important part of this signalling pathway. In the heart, a rise in cAMP levels induces an increase in contractility (inotropy), beating frequency (chronotropy), relaxation (lusitropy), excitability (bathmotropy) and conductivity (dromotropy). As a mediator of a large and diverse number of biological functions, cAMP signalling is highly compartmentalised in cells and proteins of this signalling pathway form a large number of different protein complexes (Fig. 1). One of the functions of these protein complexes is to allow spatiotemporal control of cAMP production, which is thought to be important to achieve ligand- and cell-specific responses (Musheshe et al. 2018). It is thought that cAMP does not freely diffuse in cells but is locally synthesized and degraded. Thus, this signalling pathway involves the generation of cAMP nanodomains and alterations in size and subcellular distribution of these nanodomains is associated with heart disease (Bastug-Ozel et al. 2019).

### β-adrenoceptors and adenylyl cyclases

Adrenergic signalling is initiated via β-adrenoceptors (β-AR) located in the plasma membrane. While there is evidence for three β-AR isoforms (β1, β2 and β3) expressed in the heart, most work in relation to cAMP signalling has focussed on β1- and β2-AR. β1- and β2-AR differ in their subcellular localisation initiating different physiological responses. In rodent ventricular myocytes, β1-ARs are evenly distributed throughout the plasma membrane, while β2-ARs are confined to the T-tubules, a complex tubular network of membrane invaginations reaching deep inside the cell, which allow for rapid action potential propagation and efficient excitation–contraction coupling (Nikolaev et al. 2006). Stimulation of β-ARs results in the activation of adenylyl cyclases (AC), which catalyse the synthesis of cAMP from ATP. There are 9 membrane-bound isoforms of AC (AC1-9) and one soluble isoform (sAC) (Baldwin and Dessauer 2018). In the rodent heart, AC5 and AC6 are the main isoforms. They are closely related and are activated by similar regulatory pathways but have distinct functions and differ in their subcellular localisation (Scott et al. 2013). Their molecular interplay is complex but put simply, as deducted by Baldwin et al., in mice AC5 is largely associated with stress responses and AC6 is necessary for Ca^2+^-handling and contractility (Baldwin and Dessauer 2018). Interestingly, cells in the sinoatrial node (SAN) of the rabbit and guinea pig additionally express notably high levels of AC1 and AC8, which are both Ca^2+^-activated (Mattick et al. 2007; Younes et al. 2008). Overexpression of AC8 in transgenic mice leads to increased heart rate and reduced heart rate variability (Matt et al. 2015). However, abnormal heart rate was not reported for either *Adcy1* or *Adcy8* null mutants, which is possibly due to functional redundancy of these two isoforms (Watson et al. 2000; Wu et al. 1995). AC2, AC4 and AC9 are also expressed at low levels in the heart (Baldwin and Dessauer 2018). Interestingly, in cardiac myocytes, AC9 forms a complex with the A kinase anchor protein (AKAP) Yotatio (AKAP9), Potassium Voltage-Gated Channel Subfamily Q Member 1 (KCNQ1) and protein phosphatase 1 (PP1) (Marx et al. 2002; Li et al. 2012). KCNQ1 is the pore forming subunit of a potassium channel responsible for I_ks_, a slow outward current important for cardiac repolarisation. Examination of an *Adcy9* null mutant strain revealed a sinus bradycardia and a diastolic dysfunction with preserved ejection fraction (Li et al. 2017). These data suggest that AC9 may be essential for cardiac pacemaking.

### Phosphodiesterases

Cyclic nucleotide phosphodiesterases (PDEs) catalyse the degradation of cAMP and cGMP to AMP and GMP, respectively. The PDE superfamily consists of 11 different PDE families (PDE1-11), which are encoded by 21 different genes. Each PDE gene generates multiple isoforms through alternative splicing or the utilization of alternative transcription start sites. These isoforms differ in their subcellular localisation, interaction partners, affinity for cyclic nucleotides, enzyme kinetics and regulation (Conti et al. 2014). The PDE families can also be grouped by their substrate specificity: PDE1, 2, 3, 10, and 11 are dual-specific and degrade both cAMP and cGMP, PDE4, 7 and 8 are cAMP-specific, while PDE5, 6 and 9 degrade cGMP (Bender and Beavo 2006). While at least five PDE families (PDE1-5) modulate cAMP levels in the ventricle, PDE3 and PDE4 represent the major cAMP PDE enzyme families (Mongillo et al. 2004; Wechsler et al. 2002). There are species-specific and also probably chamber-specific (atrium vs. ventricle) differences in the PDE isoforms that are predominantly expressed. In the mouse, PDE4 contributes to about 60% of overall PDE activity, while PDE3 accounts only for approximately 30%. In contrast, in the human heart PDE3 is predominant and together with PDE1 and PDE2 makes up 90% of the overall PDE activity. PDE5 is also expressed in the heart and PDE5 inhibition appears to be beneficial for patients with myocardial infarction preventing the transition to heart failure (Takimoto et al. 2005; Lawless et al. 2019). PDE3 inhibition in the human and PDE3 or PDE4 in the mouse increase the basal SAN beating frequency (Hua et al. 2012; Jaski et al. 1985). However, adrenergic stimulation of cardiac pacemaking is not affected by blocking either PDE3 or PDE4, which suggests that the catecholamine-induced increase in pacemaker activity is independent of these PDE isoforms (Galindo-Tovar and Kaumann 2008). It is therefore interesting that PDE1A is expressed at high levels in the rabbit SAN and possibly important for the adrenergic control of cardiac pacemaking (Lukyanenko et al. 2016).

### Effector proteins of the cAMP signalling pathway

The established cAMP effector proteins are protein kinase A (PKA) (Taylor et al. 2012), cyclic nucleotide-gated ion channels such as the hyperpolarization-activated cyclic nucleotide-gated (HCN) channels (Ludwig et al. 1998) and the exchange factor directly activated by cAMP (EPAC) (de Rooij et al. 1998; Kawasaki et al. 1998). Another cAMP effector protein, which is largely confined to spermatocytes and named cyclic nucleotide receptor involved in sperm function (CRIS) is involved in spermiogenesis and the control of flagellar bending (Krahling et al. 2013).

PKA is a tetramer consisting of two regulatory (R) and two catalytic (C) subunits. There are three isoforms of the catalytic subunit called Cα, Cβ and Cγ and two classes of R-subunits, RI and RII, which are subdivided into α and β subtypes (Ilouz et al. 2017; Cadd and McKnight 1989). Each isoform is encoded by a unique gene and preferentially expressed in different cells and tissues. RIα and RIIα are ubiquitously expressed in every cell, whereas RIβ and RIIβ display a more tissue restricted expression pattern (Cadd and McKnight 1989).

EPAC proteins are guanine-nucleotide exchange factors for the rat sarcoma (Ras)-like GTPases, Ras-related protein (Rap)1 and Rap2 (de Rooij et al. 1998; Kawasaki et al. 1998). In mammals, two EPAC isoforms, EPAC1 and EPAC2 control Ca^2+^-homeostasis and hypertrophy in cardiac myocytes. Interestingly, both isoforms differ in their subcellular localization. EPAC2 is mostly present at the T-tubules and EPAC1 is localized perinuclear (Pereira et al. 2015). EPAC1 is also present in mitochondria and EPAC1 null mutants display a reduction in infarct size in response to myocardial ischemia and reperfusion (Fazal et al. 2017).

The HCN genes encode nonselective voltage-gated cation channels, which are responsible for the funny current I_f_ and are largely confined to pacemaker tissue present in the SAN and atrioventricular nodes (AVN) and in the ventricular conduction system (Herrmann et al. 2011). Upon binding of cAMP, the HCN4 channel opens more rapidly and completely (DiFrancesco and Tortora 1991). This property has led to the assumption that the cAMP-mediated enhancement of HCN channel activity is largely responsible for the increase in heart rate in response to β-adrenergic stimulation. However, the *Hcn4* knockout phenotype suggests a role as a backup for cardiac depolarization rather than being essential for heart rate adaptation to stress (Herrmann et al. 2007).

### AKAP proteins and cAMP nanodomains

Protein kinase A and EPAC do not freely diffuse through the cell but are bound to different subcellular compartments. This is achieved by A kinase anchoring proteins (AKAPs) (Scott and Santana 2010). It is estimated that there are > 40 different AKAPs, which are structurally diverse. However, a unifying principle is the presence of a 14–18 amino acids long protein kinase A binding domain, which consists of an amphipathic helix, which binds to the regulatory subunits of PKA (Scott et al. 2013). The majority of AKAPs bind to the PKA-RII isoform due to structural differences between PKA-RI and PKA-RII. However, some AKAPs such D-AKAP-1/2 recognize the PKA-RI isoform (Autenrieth et al. 2016). Since the presence of the AKAP helix is the only requirement for an AKAP protein, there is a large number of proteins that serve as an AKAP acting in many different subcellular contexts. Approximately 15 AKAPs have been identified in the heart, which play important roles in a number of pathways such as calcium-induced calcium release (AKAP18α, AKAP18γ, AKAP79), repolarization (Yotatio, D-AKAP2), and stress-response (AKAP-Lbc, mAKAPβ, D-AKAP1) (Ercu and Klussmann 2018). AKAPs not only bind PKA, but also other elements of the cAMP signalling pathway including other effector proteins such as EPAC, AC and PDE isoforms as well as proteins of other signalling pathways (Dodge-Kafka et al. 2005).

## The Popeye domain containing proteins—a novel class of cAMP effector proteins

**Fig. 2:**
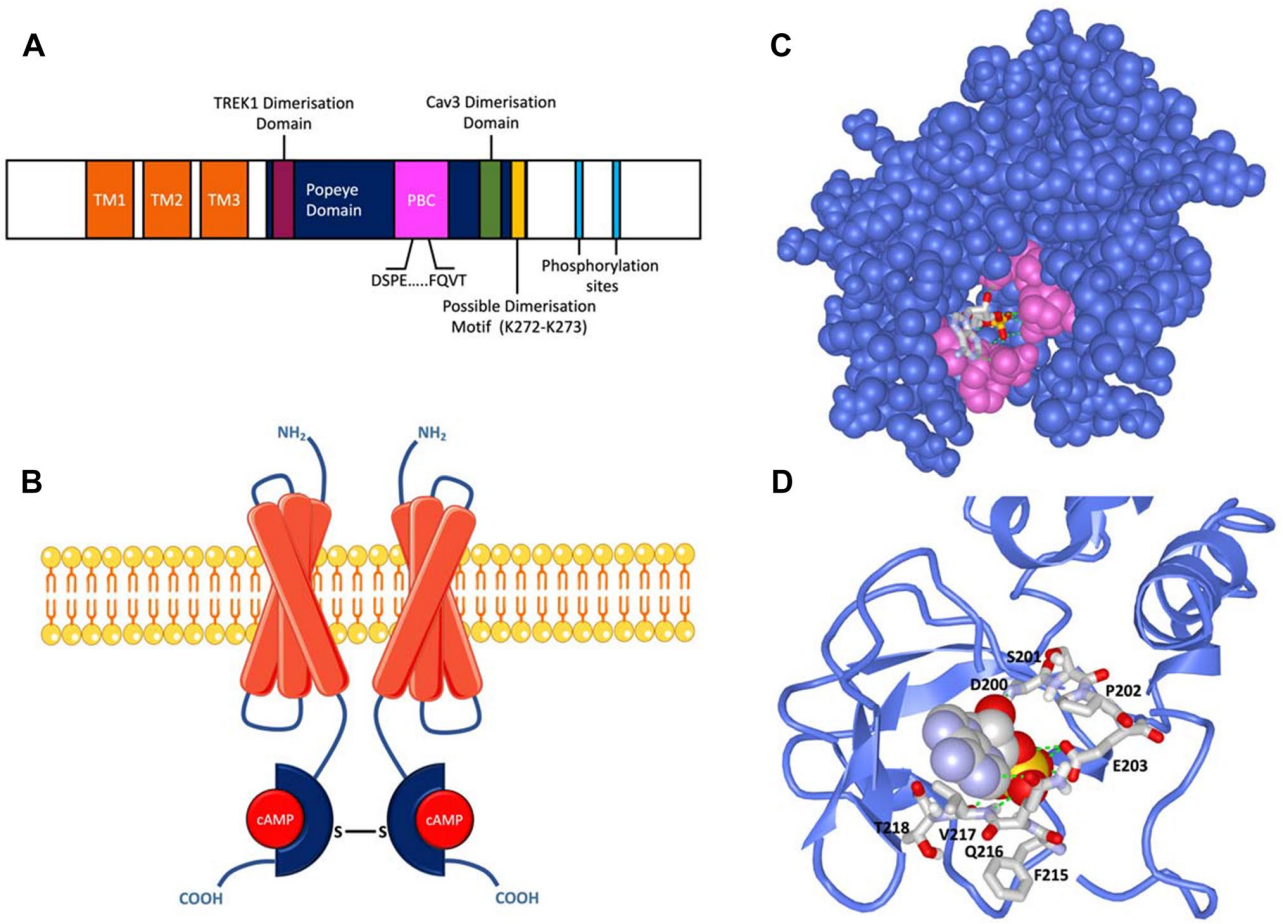
Structure and Function of the Popeye domain containing proteins. **a** Schematic of the general, linear structure of the POPDC proteins. The short amino terminus contains one (POPDC2 or POPDC3) or two (POPDC) N-glycosylation sites. Three transmembrane domains (TM) are followed by the intracellular Popeye domain, which contains the PBC, including the conserved DSPE and FQVT motifs. The putative POPDC dimerization motif (K272/K273) (Kawaguchi et al. 2008), is shown along with the proposed interaction sites of TREK1 (Schindler et al. 2016) and CAV3 (Alcalay et al. 2013). The carboxy terminus contains a number of conserved phosphorylation sites, which are subject to phosphorylation in response to adrenergic stimulation (Lundby et al. 2013). **b** A schematic of the quaternary structure of a membrane associated POPDC dimer. Each Popeye domain is shown bound to a molecule of cAMP (red sphere). In case of POPDC1 an intramolecular disulphide bridge stabilises the homodimer. **c** A homology model of the Popeye domain of POPDC1. The conserved DSPE and FQVT motifs, part of the proposed PBC, are shown in pink, with a molecule of cAMP bound. **d** An enlargement of the cAMP bound PBC from the model shown in **c**. The position of the DSPE and FQVT motifs are indicated. The models in C and D were created using the Phyre 2 algorithm (Kelley et al. 2015) and the image was rendered using iCn3D (iCn3D 2016)

The POPDC proteins share common structural features. They are medium-sized with isoforms containing between 290 and 360 residues (Andrée et al. 2000). They are integral membrane proteins with three transmembrane domains (Knight et al. 2003) (Fig. 2). The amino terminus of the protein is extracellularly localized and only 20–40 residues short, however, one (POPDC2 and POPDC3) or two (POPDC1) functional N-glycosylation sites are present (Knight et al. 2003). The extent of glycosylation appears to be tissue-specific (Vasavada et al. 2004). The cytosolic portion of the protein entails the characteristic and highly conserved Popeye domain, followed by the carboxyterminal domain (CTD), which is variable in length and isoform-specific (Andrée et al. 2000). Native POPDC1 proteins can be extracted as protein dimer (Vasavada et al. 2004) and two lysine residues (K272/K273) have been shown to be required for its formation (Kawaguchi et al. 2008).

In 2012, Froese et al. reported that the Popeye domains of POPDC isoforms function as high-affinity binding sites for cAMP (Froese et al. 2012). The predicted secondary structure of the Popeye domain revealed structural similarities with the cyclic nucleotide binding domain (CNBD) of the classical cAMP effector PKA. However, the primary sequence of the proposed phosphate binding cassette (PBC), an essential feature of CNBDs, was divergent from that of PKA (Froese et al. 2012). CNBDs are comprised of a PBC, a helical bundle at the N-terminus of the CNBD, and a C-terminal hinge and lid region (Berman et al. 2005; Rehmann et al. 2007). CNBDs are described as having a jelly-roll β-barrel fold, however this is not a unique feature of CNBDs (Kannan et al. 2007). The PBC, which contains a short loop and an α-helix between two β-sheets, makes direct interactions with the cyclic nucleotide (Berman et al. 2005). In classical CNBDs, the binding of a cyclic nucleotide induces a re-orientation of the PBC. This releases steric strain and facilitates a conformational shift in the hinge and lid region, which is further transduced to the helical bundle. The lid is then able to interact with the adenosine base of cAMP, further stabilising the bound conformation. The position of the lid is usually a key driver in the transduction of downstream events after cAMP binding (Rehmann et al. 2006).

Several proteins that share a similar sequence to classical CNBDs do not specifically bind cyclic nucleotides (Kannan et al. 2007). Given the atypical nature of the proposed CNBD function of the Popeye domain, strong empirical evidence was required to be confident that the Popeye domain was truly acting as a CNBD. cAMP binding was first demonstrated through affinity precipitation and dose-dependent elution from cAMP-agarose beads across all three POPDC protein family members (Froese et al. 2012) A competitive radio-ligand binding assay using a recombinant Popeye domain showed inhibition of unlabelled cAMP binding with an IC_50_ of 118 nM, comparable to the cAMP binding affinity of PKA. It was found that cAMP binds with 40-fold higher affinity than cGMP (IC_50_ = 5.28 µM), which is also similar to that of the CNBD of PKA (Lorenz et al. 2017). The high cAMP binding affinity was subsequently corroborated using a bimolecular Foerster resonance energy transfer (FRET) sensor based on the protein–protein interaction of POPDC1 and the two-pore potassium channel TREK-1 (Froese et al. 2012).

Insights into the binding mode of cAMP to classical CNBDs were mainly achieved via protein crystallography (Rehmann et al. 2007). The lack of a crystallographic structure of the POPDC protein, or even of the isolated Popeye domain, means that the exact mode of binding of cAMP to the Popeye domain remains unclear. The divergence of the proposed CNBD of POPDC proteins from the classical CNBDs of PKA and EPAC adds a further challenge. Other known CNBDs, such as those of the regulatory subunit II (PKA RII, PDB entry: 1CX4) and the N terminal domain (NTD) of a transcriptional regulator found in *Streptomyces coelicolor* (PDB entry: 2PQQ) have been used as templates for homology modelling of the Popeye domain (Froese et al. 2012). These models predicted the presence of a α-helical lid region and adjacent β-sheets, resembling, to some extent, a classical CNBD. cAMP docking experiments were run to predict the mode of cAMP binding (Froese et al. 2012). Two conserved motifs, FL/IDSPEW/F and FQVT/S, which are found in the Popeye domain of all three family members were predicted to form an atypical PBC and to be directly involved in cAMP binding (Froese et al. 2012). The validity of these predictions was then assessed using charge-to-alanine mutations. Indeed, a D200A mutation of POPDC1 (part of the FL/IDSPEW/F motif) was found to result in a 90% reduction in cAMP binding ability (Froese et al. 2012). E203A and V217F variants also showed a significant reduction in cAMP binding affinity. Further support for the view that the FL/IDSPEW/F motif is directly involved in cyclic nucleotide binding is based on the identification of a *POPDC1* p.S201F mutation, which has been identified in patients with muscular dystrophy and cardiac arrhythmia phenotypes (Schindler et al. 2016). The POPDC1^S201F^ mutant protein displays approximately 50% less cAMP binding affinity. S201 possibly forms a hydrogen bond between its hydroxyl group and 2′-OH group of the ribose ring of cAMP. The loss of this interaction, along with the increased steric hindrance of the larger phenylalanine residue may explain the reduction in cAMP binding affinity of the S201F mutant. Although the proline residue P202, which is part of the FL/IDSPEW/F motif) is strongly conserved, the P202A mutation had no effect on the cAMP binding ability of POPDC1 (Froese et al. 2012).

**Fig. 3:**
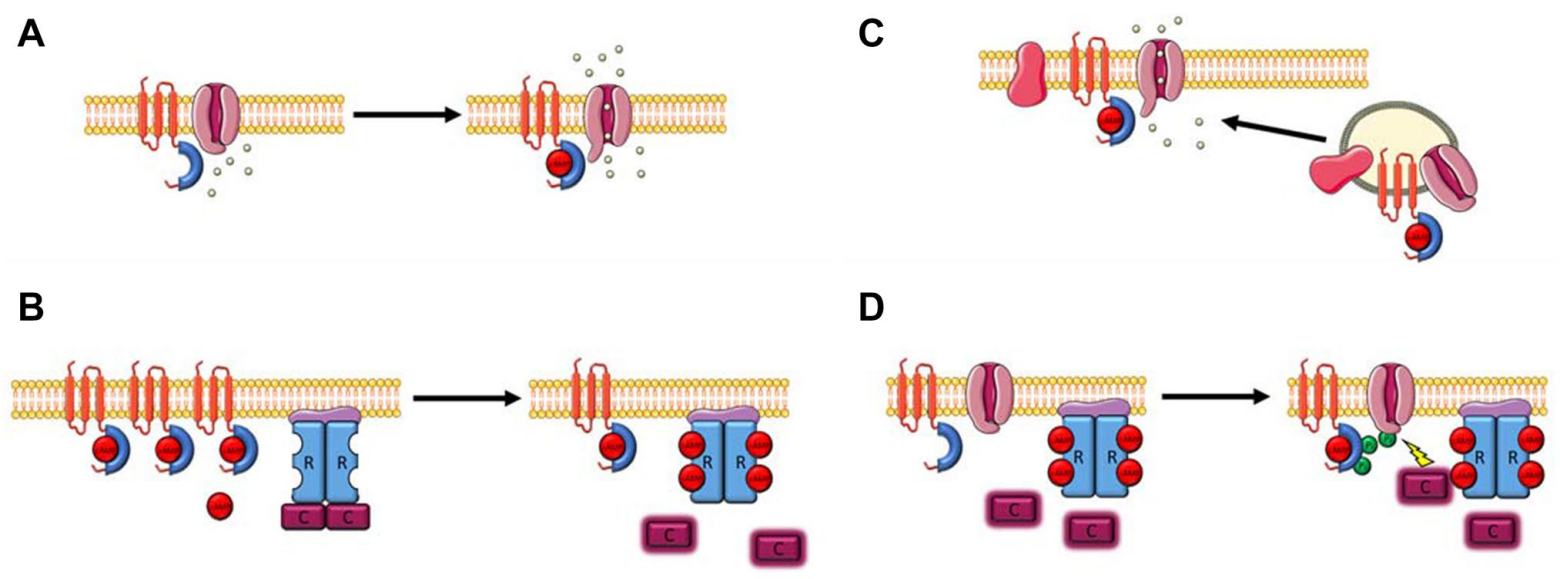
Four proposed working models of the role of POPDC proteins in the cAMP pathway. **a** The switch model. Binding of cAMP to POPDC leads to a direct change in activity of an interaction partner e.g. opening of TREK1. **b** The sponge model. The binding of cAMP to POPDC proteins leads to a reduction in the local cAMP concentration. This reduces activation of other proteins in the cAMP pathway, in this case PKA. A lowering of POPDC expression (such as in null mutants), or alternatively a reduction in the cAMP binding affinity (such as in missense mutations found in patients), leads to an increase in the free cAMP concentration and therefore results in stronger and more sustained activation of cAMP effector proteins. **c** Cargo model. This model proposes that cAMP influences the POPDC protein’s role in modulating membrane influences the POPDC protein’s role in modulating membrane expression of interaction partners. The exact effect of cAMP binding on vesicle transport and membrane trafficking is currently not fully understood. **d** The shield model. This model builds on the switch model and suggests that cAMP binding to the Popeye domain may lead to indirect downstream effects, possibly via phosphorylation of the POPDC proteins. For example, in the unbound state POPDC proteins may shield interaction partners from being accessed by kinases, with this inhibition being reduced after cAMP binding to the Popeye domain. How the phosphorylation and conformational changes of POPDC proteins after cAMP mediate downstream effects is still unclear

The binding of cAMP to classical cAMP effectors results in some acute behavioural change in the protein. For example cAMP binding to the regulatory subunits of PKA leads to the dissociation of, and subsequent activation, of the catalytic subunits from the regulatory subunits (Walker-Gray et al. 2017). In case of the Popeye domain, any conformational changes induced upon binding to cAMP, such as those in classical CNBDs, have not been empirically verified. The lack of crystallography or NMR-derived structures of the Popeye domain has proved to be a major hindrance in this respect. However, some downstream effects of cAMP binding to POPDC have been studied, notably the interaction with the cardiac two-pore potassium channel, TREK-1. The binding site for TREK-1 has been mapped to the beginning of the Popeye domain (Fig. 2). A FRET assay utilising CFP-tagged POPDC1 and YFP-tagged TREK-1, co-expressed in HEK293A cells, was used to study the cAMP dependency of the interaction (Froese et al. 2012; Schindler et al. 2016). When the intracellular cAMP concentration was raised, either through application of isoproterenol or forskolin, an acute, dose-dependent reduction in FRET signal was observed. Further, when D200A and S201F variants of POPDC1, which respectively display around a 90% and 50% reduction in cAMP affinity compared to wild-type POPDC1 protein, were co-expressed with TREK-1, the change in FRET signal after cAMP stimulation was significantly reduced (Froese et al. 2012; Schindler et al. 2016). The lack of response to nitroprusside, which raises intracellular cGMP concentrations, further demonstrates the selectivity for cAMP over cGMP under physiological conditions. Co-expression with POPDC1 significantly increased TREK-1 plasma membrane expression in HEK293 cells and also raised the TREK-1-dependent outward K^+^ current. Long-term incubation of cells co-expressing TREK-1 and wild-type POPDC1 in media containing 1 mM 8-Br-cAMP led to a reduction of the TREK-1 current; whereas in cells expressing TREK-1 and POPDC1^S201F^, the TREK-1 current was insensitive to the presence of 8-Br-cAMP and showed no immediate change (Froese et al. 2012; Schindler et al. 2016). This is interesting as the FRET assay showed an immediate change in the TREK-1-POPDC1 interaction after application of isoproterenol, but patch clamp experiments suggest that this has no immediate effect on the current, suggesting that POPDC1 may not act as a switch (see Fig. 3 for a working model) to in order to modulate the gating properties of TREK-1. However, the results could also be explained by the modest membrane permeability of 8-Br-cAMP, leading to low intracellular concentrations during the short time span of the experiment. This experiment therefore needs to be repeated with alternative cyclic nucleotide derivatives, possessing a higher membrane permeability while simultaneously monitoring cAMP levels using FRET sensors (Bartsch et al. 2003). This interaction may be important as it is thought that POPDC1 has a modulatory effect on TREK-1, and dysregulation of this interaction may contribute to the cardiac arrhythmia phenotypes observed in *POPDC* knock-in (KI) and knockout (KO) mutants.

## Animal models

### A stress-induced bradycardia in Popdc1 and Popdc2 null mutants

POPDC proteins are highly expressed in striated muscle, however, our knowledge of their function and regulation is still limited. To this end *Popdc1* and *Popdc2* knockout mouse models were generated by replacing the first coding exon of each gene with a nuclear LacZ reporter gene (Andrée et al. 2002a; Froese et al. 2012). Both null mutants were viable and did not display any embryonic lethality. Analysis of the LacZ reporter gene expression revealed that *Popdc1* and* Popdc2* are expressed in an overlapping manner in the mouse heart (Andrée et al. 2002a; Froese and Brand 2008; Froese et al. 2012). While each POPDC isoform is expressed in the heart, the expression domains are not identical. Thus, while *Popdc2* is expressed homogeneously in both atrial and ventricular myocardium, *Popdc1* is expressed at higher levels in the atria in both chicken and mouse hearts (Froese et al. 2012; Torlopp et al. 2006). Both Popdc1 and Popdc2 share high expression levels in the SAN and AVN and also in the ventricular conduction system (Froese et al. 2012). Chamber-specific differences have also been reported for the human heart (Gingold-Belfer et al. 2011).

*Popdc1* and *Popdc2* null mutant mice did not reveal any cardiac pathology under resting conditions. However, both mutants showed extensive sinus pauses and episodes of tachybradycardia with increased heart rate variability and reduced mean heart rate in response to emotional and physical stress as well as in response to an injection of the β-AR agonist isoproterenol (Froese et al. 2012).

Pacemaker cells are a primitive form of cardiac myocyte which are responsible for triggering a regular heartbeat and are able to adapt the frequency to the physiological demand. An important current for pacemaking is I_f_ or funny current (I_f_). This current is mainly produced by HCN4 an excellent marker for SAN development, structure and function. HCN4 is uniquely expressed in the SAN and AVN and a major determinant of cardiac pacemaking (DiFrancesco 2010; Monfredi et al. 2013). I_f_ in collaboration with a number of other sarcolemmal ion channels and pumps (membrane clock), interacts with the so-called calcium clock. This involves the sarcoplasmic reticulum (SR)-localised Ca^2+^-release channel ryanodine receptor (RYR2) and the sarcoplasmic/endoplasmic Ca^2+^-ATPase (SERCA II), which are responsible for local Ca^2+^ release (LCR) and reuptake into the SR, respectively. The submembraneous LCR triggers a sodium current (I_NCX_) driven by the Na^+^/Ca^2+^ exchanger (NCX), which helps to depolarise the pacemaker cell and elicit an action potential. Currently, it is unclear if POPDC proteins interact directly or indirectly with elements of the Ca^2+^- or membrane clock. In *Popdc2* null mutants, I_f_ was found to be unaltered, however, it was recently reported that POPDC2 directly interacts with the NCX1 (Froese et al. 2012; Lubelwana Hafver et al. 2017).

Whole mount immunohistochemical staining, utilising HCN4 as an SAN marker, of sinoatrial preparations from wild-type and *Popdc1* and *Popdc2* mutants revealed dramatic changes in SAN structure in 8 months old mutant hearts but not in 3 months old mutants. A distinct change in cell morphology was observed in mutant cells (Froese et al. 2012). Pacemaker myocytes in healthy specimens have long, thin cell extensions and are termed spider or spindle cells (Wu et al. 2001). In tissue samples of *Popdc1* and *Popdc2* knockout mice fewer of these cells were present, which may become a limiting factor for cardiac pacemaking under stress. Moreover, the lack of cellular extensions in nodal myocytes of *Popdc1* or *Popdc2* null mutants may also lead to an impaired electrical conduction from the SAN to the atrial myocardium (Opthof et al. 1987). Furthermore, the HCN4 staining was also indicative of a more compact SAN structure overall with a loss of cells in the inferior part of the node. These cells are particularly important during beta-adrenergic signalling due to a shift of the primary pacemaker location to the inferior SAN (Opthof et al. 1987). Interestingly, the structural changes, as well as the mutant phenotype, were only apparent in mutants aged 5 months or older, indicating that the observed changes are age-dependent (Froese et al. 2012). However, at present it is not known whether the structural changes are a consequence of the underlying electrical changes or whether the structural changes lead to the observed changes in conduction.

### *Popdc1* null mutants display an increased ischemia–reperfusion injury

Caveolae are specialised membrane invaginations that are enriched with cholesterol and glycosphingolipid and are structurally stabilised by scaffolding proteins called caveolin (CAV) (Vaidyanathan et al. 2018). In striated muscle cells, CAV3 is the main scaffolding protein isoform. Caveolae are thought to be important for compartmentalization of the sarcolemma and both β-AR, AC and several protein pumps and exchangers (NCX), ion channels (inward rectifier potassium, sodium and L-type calcium channels) are found in caveolae. These membrane structures create cellular microdomains providing confined environments important for precise regulation of and crosstalk between signalling pathways. Recently it has been reported that POPDC1 is localised in caveolae and its presence is critical for cardiac protection and ischemic preconditioning (Alcalay et al. 2013). POPDC1 interacts with CAV3 via its consensus sequence found at the end of the Popeye domain (Fig. 2). In *Popdc1* null mutants, the number of caveolae is dramatically reduced, while an increase in size of the remaining rafts was observed (Alcalay et al. 2013). Interestingly, *Popdc1* null mutant cardiac myocytes display impaired calcium transients, an increase in oxidative stress sensitivity and an impaired preconditioning response. Additionally, *Popdc1* mutant hearts display an increased susceptibility to ischemia/reperfusion (I/R) injury (Alcalay et al. 2013). Moreover, retrograde Langendorff perfusion experiments revealed that *Popdc1* mutant hearts develop larger infarct sizes suggesting that POPDC1 might have a protective function in cardiac myocytes.

### POPDC morphants and mutants in zebrafish develop cardiac arrhythmia and muscular dystrophy

Expression analysis of the three POPDC genes in zebrafish revealed a strong association with striated muscle (Kirchmaier et al. 2012). Morpholino-mediated knockdown of *popdc2* induced a severe muscular dystrophy phenotype in 5-day old zebrafish larvae. A similar phenotype was present in *popdc1* morphants and in *popdc1*^S201F^ knock-in mutants (Schindler et al. 2016). Many mutants and morphants developed a pericardial effusion (Kirchmaier et al. 2012; Schindler et al. 2016). Pericardial effusion is often interpreted as a sign of the presence of embryonic heart failure. However, impaired tight junction formation might be causing a defective barrier function of the *popdc1* morphants’ skin resulting in water influx, pericardial effusion and generalized oedema formation (Wu et al. 2012). Interestingly, the oedema formation was rescued when morphants were kept under hyperosmotic conditions. Morphants and *popdc1*^S201F^ KI mutants also developed an AV-block during larval development, which was characterized by an age-dependent increase in phenotype severity (Kirchmaier et al. 2012; Schindler et al. 2016).

## Mutations in POPDC genes in patients are associated with muscular dystrophy and cardiac arrhythmia

Several patients have been discovered to carry mutations in *POPDC1*. These patients suffer from limb girdle muscular dystrophy (LGMD) and cardiac arrhythmia (De Ridder et al. 2019; Schindler et al. 2016). However, there is huge phenotypic variability associated with *POPDC1* mutations. Symptoms that are commonly present in these patients are muscle weakness, elevated serum creatine kinase (CK) levels and atrioventricular (AV) block. However, the AV-block was either nocturnal or persistent, a mild first-degree AV-block, or as severe as a complete heart block (De Ridder et al. 2019; Schindler et al. 2016). The most well-studied POPDC mutation is the *POPDC1*^*S201F*^ mutation, a recessive point mutation, which affects one of the invariant amino acids present in the putative cAMP binding domain (DSPE motif). Measurement of cAMP binding revealed a 50% reduction in the mutant protein (Schindler et al. 2016). Three family members (grandfather and two grandsons), are homozygous for the mutant allele and are suffering from the disease. The affected grandfather developed a late onset LGMD, which began in his 40 s and resulted in a loss of ambulation by the age of 60. Both grandsons suffer from syncopal episodes, which began in their adolescence. Both display type II AV block, while one also shows sinus bradycardia (Schindler et al. 2016). Although both grandsons have elevated CK levels, neither developed a muscular dystrophy suggesting that the LGMD is a late onset feature of the disease. Recently, another three recessive mutations in *POPDC1* have been discovered. An internal deletion (*POPDC1*^del56V217-K272^) due to a splice acceptor site mutation, a *POPDC1*^R88X^ nonsense mutation and a presumptive null mutation due to the presence of an A>C mutation in the start codon (De Ridder et al. 2019; Schindler et al. 2016).

Immunohistochemical staining of muscle biopsy material obtained from patients carrying one of the mentioned mutant alleles of *POPDC1* with antibodies for POPDC1 and POPDC2 revealed a loss of membrane localization of the mutant POPDC1 protein (De Ridder et al. 2019; Schindler et al. 2016). Interestingly, defective membrane trafficking was also observed for POPDC2. Thus, the underlying cellular pathology, i.e. impaired membrane trafficking of POPDC1 and POPDC2 was invariably present in each patient carrying one of the four discovered *POPDC1* mutations, which is in contrast to the variable clinical features observed in this patient population.

A heterozygous nonsense mutation in *POPDC2* has been recently identified in two families suffering from first-, second-, or third-degree AV block (Rinné et al. 2016). The mutation generates a premature stop codon at position 188 resulting in a truncated *POPDC2*^*W188X*^ protein with a partial loss of the Popeye domain. Surprisingly, the truncated protein retained its ability to bind cAMP with a similar affinity as the wild-type protein and was still able to interact with the two-pore potassium channel TREK1 and the cardiac sodium channel SCN5A. Co-expression of *POPDC2*^*W188X*^ and TREK-1 or SCN5A in *Xenopus* oocytes revealed an aberrant current modulation by the mutant protein.

Genome wide association studies (GWAS) utilized epigenomic signatures to validate subthreshold loci associated with long QT syndrome (Wang et al. 2016). One of the novel loci identified in this way affects a cardiac enhancer that controls the expression level of *POPDC1* and *POPDC3* and the SNP causes impaired binding of nuclear factor I binding. These data suggest that mutations affecting the expression level of *POPDC1* might also give rise to long QT syndrome.

As more mutations in POPDC gene are discovered, a disease spectrum emerges. In the heart, mutations of *POPDC1* or -2 affects AV conduction while a structural defect (LGMD) is seen in skeletal muscle. *POPDC1* mutations are associated with both heart and skeletal muscle defects, while *POPDC2* mutations only affect the heart. This difference between these two genes may be based on the postnatal expression pattern as *POPDC1* is expressed equally in both skeletal muscle and heart, while *POPDC2* is expressed at much higher levels in the heart (Andrée et al. 2000).

## POPDC1 acts as tumour suppressor

It has long been known that POPDC1 is required for the formation and maintenance of tight junctions (Osler et al. 2005; Russ et al. 2011), and more recently has been associated with adhesion junctions in certain tissues (Williams et al. 2011). Due to POPDC1’s involvement in these junctions, it is not surprising that its loss has been associated with epithelial-to-mesenchymal transition (EMT) (Han et al. 2014; Williams et al. 2011). Several cancers feature an improper EMT regulation (Heerboth et al. 2015; Wang et al. 2011) and downregulation of epithelial marker genes often indicates a poor prognosis for patients as the cancer can more easily metastasize (Thomas et al. 2005).

All three POPDC family members have been implicated in different forms of cancer. However, *POPDC1* has been most intensively studied and downregulation was shown in breast, lung liver and colorectal tumours (Amunjela and Tucker 2017b; Han et al. 2015; Han et al. 2014; Parang et al. 2017; Williams et al. 2011). In several of these tumours *POPDC1* is hypermethylated, which causes a reduction in its expression level. The molecular mechanisms of how a reduction in *POPDC1* expression promotes malignant tumour growth is still unknown. However, a molecular link to c-Myc (Parang et al. 2017) and the WNT co-receptor LRP6 (Thompson et al. 2019) has recently been established.

c-Myc is a proto-oncogene that has been shown to be upregulated in many forms of cancer (Chadd et al. 1999). Its downstream target genes are involved in cell cycle control and cell proliferation and therefore stabilizing c-Myc levels promotes tumour growth (Emmett 1999). However, in healthy tissues, *c*-*Myc* expression and protein activity is under tight regulation in order to maintain homeostasis (Lang et al. 1988; Vervoorts et al. 2006). One of these rigorous control mechanisms involves the phosphorylation of c-Myc at different aminoterminal residues (Sears et al. 2000). Through phosphorylation at serine 62 (S62), by mitogen-activated protein (MAP) kinase (Alvarez et al. 1991; Lutterbach and Hann 1994), the cytoplasmic level of c-Myc is stabilised. However, S62 phosphorylation is also required for the phosphorylation of threonine 58 (T58) (Arnold and Sears 2006; Sears et al. 2000; Yeh et al. 2004) by glycogen synthase kinase 3 alpha (GSK3α) (Lutterbach and Hann 1994). Phosphorylation of T58 destabilises c-Myc by enabling the PR61α subunit (also known as B56a) of protein phosphatase 2A (PP2A) to dephosphorylate c-Myc at S62 (Arnold and Sears 2006), which ultimately leads to the ubiquitination of c-Myc and its targeting for the proteasomal degradation pathway (Sears et al. 2000).

POPDC1 interacts with the PR61α subunit of PP2A along with c-Myc itself (Parang et al. 2017). Furthermore, POPDC1 has been shown to be downregulated in colon cancer (Williams et al. 2011), furthering the claim that POPDC1 is aiding PP2A in its negative regulation of c-Myc. This novel role of POPDC1 could turn out to be critical for the development of novel therapies for cancer patients. POPDC proteins are expressed strongly in the heart and skeletal muscle. It is therefore possible that POPDC1 is also controlling c-Myc levels in heart and skeletal muscle and thereby may modulate the regenerative potential in these tissues.

Recently, POPDC1 has also been implicated as an inhibitor of the WNT3a signalling pathway in the gut (Thompson et al. 2019). It has been suggested that POPDC1 directly interacts with the LRP6 co-receptor. Significantly, expression and phosphorylation levels of LRP6 are increased in *Popdc1* null mutants, and are thought to enhance canonical WNT signalling, which is a strong driver of cancer development (Thompson et al. 2019; Zhan et al. 2017).

POPDC1’s negative control of the WNT signalling pathway may also provide novel mechanistic insight into the muscular dystrophy phenotypes present in carriers of *POPDC1* mutations and in KO mutants in zebrafish and mice. WNT signalling is known to be important for both the maintenance and differentiation of satellite cells. Canonical WNT signalling is essential in embryonic muscle development by inducing somatic myogenesis (Muensterberg et al. 1995). Furthermore, WNT signalling is upregulated after muscle injury (Brack et al. 2007; Polesskaya et al. 2003). Aberrant WNT signalling may also form the basis for the retardation of muscle regeneration in *Popdc1* KO mice (Andrée et al. 2002a).

An open question that remains to be answered is whether a link exists between the ability of POPDC proteins to bind cAMP and mediate cAMP signalling and its role in controlling cell proliferation and tumour formation. cAMP treatment of breast cancer cell lines interferes with cell migration and tissue invasion and promotes apoptosis (Bianco et al. 1997; Spina et al. 2012). Significantly, cAMP treatment causes an enhanced expression of POPDC1 and thereby might be directly involved in mediating some of the cellular responses after cAMP treatment (Amunjela and Tucker 2017b).

## Working models of POPDC protein function

The high affinity and selectivity of the Popeye domain strongly suggests an important role for the POPDC family in the cAMP signalling pathway. The pathogenicity of mutations in the proposed CNBD, such as the POPDC1^S201F^ mutation, which have no obvious other effect on the protein, adds weight to the hypothesis that cAMP binding is key to POPDC function. While the precise role of these proteins in the cAMP pathway is still uncertain, four models have been proposed (Fig. 3) (Brand and Schindler 2017). These are known as the *switch* model, the *shield* model, the *sponge* model and the *cargo* model. As already discussed for TREK-1, the switch model proposes that cAMP binding to POPDC proteins leads to a direct change in the interaction with a protein–protein interaction (PPI) partner resulting in activation or inhibition of the PPI partner’s function (Boukens and Christoffels 2012b; Brand et al. 2014). The *shield* model is an extension of the *switch* model, which describes the function of POPDC proteins not only as a cAMP effector protein, but also as a modulator of PKA-dependent phosphorylation of PPI partners. In this regard it is noteworthy that POPDC proteins are subject to extensive phosphorylation in the carboxy terminal domain (CTD) in response to β-adrenergic signalling, which by itself may lead to further downstream effects (Lundby et al. 2013). The *sponge* model describes POPDC proteins as an inhibitory check on cAMP signalling; acting by binding intracellular cAMP, limiting its diffusion and therefore participating in controlling cAMP compartmentalisation. In support of this proposed function, the expression level of POPDC proteins, particularly POPDC2, are unusually high for a membrane-bound signalling protein (Andrée et al. 2000; Froese et al. 2012). The proposed function could be experimentally tested by measuring cAMP compartmentalisation using cAMP FRET sensors (Nikolaev et al. 2005) (Surdo et al. 2017). Finally, the *cargo* model places the emphasis on the POPDC proteins’ role in membrane trafficking of its PPI partners, although how cAMP is involved in this is unclear (Brand and Schindler 2017). These models are, for now, highly simplified and it is likely that the POPDC proteins’ full role encompasses parts of each model.

## Outlook

A detailed mechanistic understanding of how the POPDC proteins carry out their many apparent functions is still unclear. Many interaction partners have now been identified, including ion channels, structural proteins and those involved in cell cycle control. However, the functional relevance of many of these interactions remains unclear. In many cases no clear link to cAMP binding to the Popeye domain has been found. The high sequence conservation of the proposed atypical CNBD within the Popeye domain throughout the animal kingdom and between isoforms suggests an important role for cAMP binding. Mutations that hinder cAMP binding have clear pathological effects. The interaction between POPDC1 and TREK-1 is the only interaction shown to be acutely altered by cAMP. The large size of the Popeye domain, relative to classical CNBDs, suggests that it does have a function beyond a cAMP binding domain, and indeed multiple PPIs are mediated via the Popeye domain (Fig. 2a). It could be that the POPDC proteins occupy distinct functional roles from that of a cAMP binding protein.

Research on POPDC protein’s role in the cAMP pathway have generally focused on cardiac and muscular phenotypes. The importance of the POPDC family in many other physiological processes such as protein trafficking, tumour cell migration and proliferation and cell viability has, so far, largely been explained independently of cAMP and implicated in, for example, the WNT signalling pathway (Thompson et al. 2019). Determining if the POPDC proteins’ place in the cAMP pathway is entirely distinct to its role mediating c-Myc or WNT signalling, or if all functions are underpinned by the binding of cAMP is an important question and may lead to a unifying concept of POPDC protein function. In an attempt to link cell cycle control and cAMP signalling function, Amunjela et al. have recently demonstrated a cAMP-mediated effect of POPDC1 on breast cancer cell line migration and proliferation (Amunjela and Tucker 2017b). Further studies into the molecular events downstream of cAMP stimulation may shed light onto the signalling pathway(s) involved.

The emerging roles of the POPDC proteins, both in cardiology and oncology, has led to interest in the family as a potential therapeutic target (Amunjela and Tucker 2016; Amunjela and Tucker 2017b; Boukens and Christoffels 2012). The clearest indications of the role of the POPDC proteins in heart and skeletal muscle diseases have come from the identification of associated missense and nonsense mutations in *POPDC1* and *POPDC2* (De Ridder et al. 2019; Rinné et al. 2016; Schindler et al. 2016). Unfortunately, the rarity of these mutations means that specifically targeting these mutations is unlikely in large demand. However, the large variability in the cardiac and muscular phenotypes observed in patient’s loss-of-function mutations in *POPDC1* means that the POPDC family may also be involved in diseases that have yet to be identified. Targeting the low expression levels in the failing heart may be a more tractable strategy (Gingold-Belfer et al. 2011). Significant reductions in expression of *POPDC1* have also been identified in multiple tumour types (Han et al. 2014; Kim et al. 2010; Luo et al. 2012; Williams et al. 2011). Indeed *POPDC1* is now thought of as a tumour suppressor gene (Parang et al. 2018). Therefore, methods of raising the expression and/or the biological activity of *POPDC1*, and maybe also *POPDC2/3*, could form the basis of a widely applicable therapeutic strategy.

The POPDC proteins possess several features which make them promising drug targets. The Popeye domain is unique to the family, showing significant divergence from the classical CNBDs. Selectivity between the various cyclic nucleotide binding proteins is a major challenge in the development of cAMP analogue-based drugs. The atypical PBC of the Popeye domain would suggest that it is likely that a selective compound could be found. In addition, the Popeye proteins are less widely expressed than CNBD containing proteins such as PKA and EPAC. This would further act to reduce off-target effects.

While the downstream effect of cAMP binding to POPDC proteins is not fully understood, the pathogenicity of mutations that lead to reductions in cAMP affinity suggest that it is key for its normal homeostatic function. Furthermore, the application of cAMP to cancer cell lines was found to upregulate expression of POPDC1, which led to the inhibition of cell migration and survival (Amunjela and Tucker 2017a). The effect of mimicking an increase of cAMP binding to POPDC in other systems is not fully understood but provides a promising line of enquiry in the hunt for POPDC protein-targeted therapies. Small molecule modulators of the POPDC proteins would be a valuable research tool, even before a therapeutic role was pursued, as it would reduce the reliance on genetic manipulation. The design of selective ligands for POPDC proteins would be greatly facilitated by an empirical structure for the Popeye domain, either from crystallography or NMR. This would also provide valuable insight into how the Popeye domain’s atypical CNBD operates.
